# Chemically Induced Breast Tumors in Rats Are Detectable in Early Stages by Contrast Enhanced Magnetic Resonance Imaging but Not by Changes in the Acute-Phase Reactants in Serum

**DOI:** 10.3390/ijms12021030

**Published:** 2011-02-07

**Authors:** Shahram Golbabapour, Wei Wei Pang, John George, Thanikachalam Pasupati, Puteri Shafinaz Abdul-Rahman, Onn Haji Hashim

**Affiliations:** 1 Department of Molecular Medicine, Faculty of Medicine, University of Malaya, Kuala Lumpur, Malaysia; E-Mails: ugreenk@gmail.com (S.G.); terisar@um.edu.my (P.S.A.-R.); 2 University of Malaya Centre for Proteomics Research, Faculty of Medicine, University of Malaya, Kuala Lumpur, Malaysia; E-Mail: wwpang@um.edu.my; 3 Department of Biomedical Imaging, Faculty of Medicine, University of Malaya, Kuala Lumpur, Malaysia; E-Mail: msk.rad@gmail.com; 4 Gribbles Pathology, Kuala Lumpur, Malaysia; E-Mail: tpasupati@gmail.com

**Keywords:** acute-phase response, breast cancer, proteomics, magnetic resonance imaging

## Abstract

The present study was undertaken to develop a rat model for monitoring the early development of breast cancer. Twelve female rats were divided into two groups of six rats that were either treated with *N*-methyl-*N*-nitrosourea to induce breast cancer or with bacterial lipopolysaccharide to induce inflammation. Serum samples taken from the rats prior to the treatment were used as controls. By the 14th week, presence of the tumor was detectable by contrast enhanced magnetic resonance imaging and confirmed by histopathology. When the serum proteins of the rats were examined by 2-dimensional electrophoresis (2-DE), no difference could be detected in the profiles of all proteins before and 18 weeks after administration of *N*-methyl-*N*-nitrosourea. However, higher expression of alpha-1B glycoprotein was detectable by 2-DE in serum samples of rats at the 18th week post-treatment with lipopolysaccharide.

## 1. Introduction

Breast cancer is one of the most common cancers in the world. It accounts for 10.9% of all cancers, with a reported incidence of about 1.38 million cases in 2008 [1]. Detection of breast cancer is heavily dependent on imaging techniques including mammograms, ultrasound, different types of scans and magnetic resonance imaging (MRI). The use of biomarkers for early diagnosis of breast cancer is not yet considered reliable. As symptoms of breast cancer widely vary and may be similar to non-cancerous conditions, like an infection or a cyst, diagnosis is typically made at the late stages of the disease.

The potential of MRI for early diagnosis of breast cancer is now recognized. The T2-weighted MRI has been reported to accurately detect high signals from rat breast tumors. Out of 18 tumors of less than 1 mm in sizes, 17 were detected without the use of contrast [2]. Dynamic contrast enhanced-MRI has also been shown to improve early detection of cancer [3–5]. A study has reported that the tumor microvessels may be recognized using the macromolecular particulate MRI contrast agent for enhancement of the reticuloendothelial system [6]. Therefore, the method may be more suitable for detection of small malignant breast tissues. In fact, the American Cancer Society has recommended the use of contrast enhanced MRI for screening of women at high risk for developing breast cancer.

Another possible approach for early breast cancer detection is by profiling changes in the acute-phase reactant proteins (APRPs) in serum. The acute-phase response is an animal’s immediate reaction to an infection or other types of immunological stress [7]. Changes in the concentrations of serum or plasma APRPs are one of the responses in an acute-phase reaction to an injury/trauma, inflammation, infection or diseases including cancer. We have been examining the APRP patterns of sera from cancer patients to evaluate their applicability for the diagnosis of cancer. By using the gel-based proteomics approach, alterations in the APRPs of sera from distinct cancers have been documented [8–11]. For example, sera from breast cancer patients showed an increased expression of three proteins and decreased expression of two proteins. Our findings are in agreement with those of many others [12–15], suggesting that the different patterns of APRP alteration may be used as protein signatures for diagnostic [16] as well as prognostic purposes [17]. Nevertheless, one deficiency of the previous studies of the APRP levels in cancer patients was that the subjects may have varied in terms of their tumor load, stages of cancer, age groups, *etc*. This was quite unavoidable since the studies were reliant on the limited availability of serum samples from newly-diagnosed and untreated patients who presented themselves at the hospital. Therefore, in order to evaluate the potential for using serum APRPs for early diagnosis of cancer an animal model will be beneficial.

In the present study, we used a rat model to investigate the early development of breast cancer using contrast enhanced MRI and determined whether changes in serum APRPs were notable in the very early stages of development of breast tumors in rats. Presence of breast tumor was confirmed by histopathological examination of the rat mammary tissues. Further, we included controls to distinguish changes that were due to inflammation *per se* as opposed to malignancy. Serum samples from rats that were chemically induced with breast cancer, as well as those induced with inflammation, were subjected to 2-dimensional electrophoresis (2-DE) and compared to the 2-DE profiles of control rat sera obtained prior to the chemical treatments.

## 2. Results

### 2.1. Histological Staining of Rat Mammary Sections

**Figure 1.**
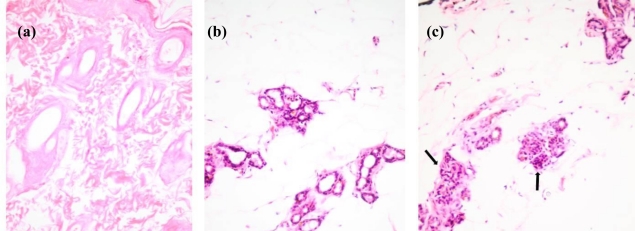
Mammary tissue histology and pathology snapshots of (**a**) normal appearance without any evidence of glandular proliferation of a normal rat, (**b**) lobular architecture of breast acini in a rat treated with bacterial lipopolysaccharide (LPS), and (**c**) ductal carcinoma *in situ* (DCIS) changes noticed in many glands (shown by arrows) in a rat treated with *N*-methyl-*N*-nitrosourea (NMU).

In contrast to the normal rat mammary tissues, the abdominal-inguinal and contralateral mammary glands of rats induced with breast cancer showed alteration in their tissue structures (Figure 1), which in comparison with the report by Murray *et al.* [18], was similar to a pregnant or lactating mammary gland phenotype. A number of alterations were observed in the morphology of breast tissues in the group of rats injected with *N*-methyl-*N*-nitrosourea (NMU). An increase in the number of acini, further epithelial differentiation with secretory material, ductal carcinoma *in situ* (DCIS), hyperplastic ducts containing more than three layers of epithelial cells and large dilated ducts in a lobular configuration were also noticed. There were occasional areas showing benign proliferation of the ducts and crowding of acini. In one of the rat samples, scattered atypical cells with hyperchromatic nuclei (an indication of early stromal invasion), in combination with DCIS was noted. This is an indication of early invasive cancerous change although there was no significant alteration that was noticed in the stroma.

### 2.2. MRI Analysis

**Figure 2.**
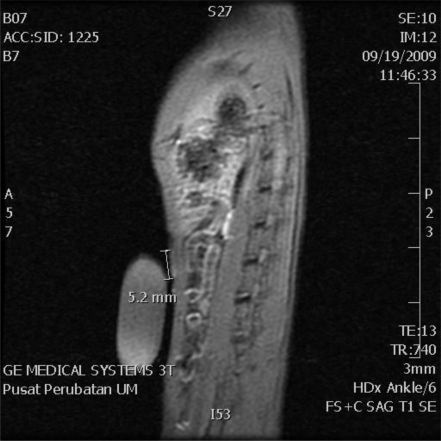
Sagittal T1 Fat Sat magnetic resonance imaging (MRI) contrast image of a rat at 14 weeks after injection of a cancerous agent. There was marked enhancement of the upper set of mammary glands: 5.2 mm in length in this rat (between small arrows) but no enhancement of the lower set of mammary glands.

Sagittal T1 Fat Sat contrast enhanced image of the rat was successfully scanned using gadolinium as a contrast agent at 14 weeks after injection of NMU (Figure 2). There was marked enhancement of the upper set of the mammary glands in the post gadolinium images measuring 2 mm in the long axis but no noticeable enhancement in the lower mammary glands.

### 2.3. 2-DE Serum Protein Profiles

**Figure 3.**
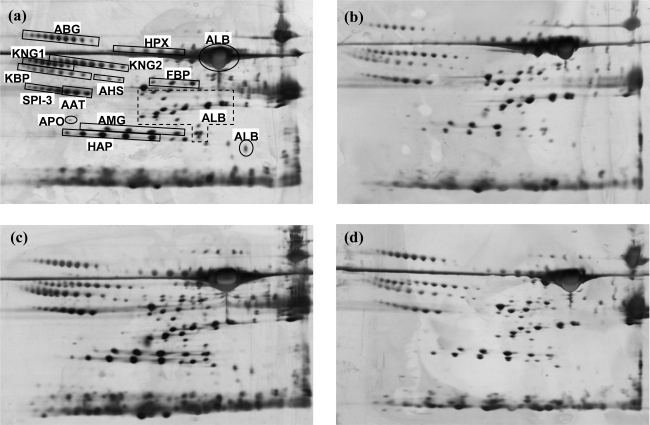
Typical 2-DE profiles of rat serum samples. Panels show typical profiles of serum proteins of (**a**) LPS-treated rats, (**b**) rats treated with NMU and their respective control sera (**c**) and (**d**). Protein spot identities and the acronyms used are in accordance to Table 1. Acid sides of gels are to the left and molecular mass declines from the top.

When proteins from control rat serum samples were separated by 2-DE and stained with silver, complex protein profiles containing hundreds of spots were obtained. Serum samples of each treated rat group were subsequently resolved using the same technique. Figure 3 shows the representative serum protein profiles of normal female control rats and rats treated with NMU and LPS, respectively. Identities of the 2-DE resolved proteins from serum isolated in the first (control samples) and the final (rats assumed to have cancer/inflammation) weeks were initially established by visual comparison of the 2-DE gels with previously published protein maps obtained from rat sera [19]. Identities of the serum proteins were then confirmed by mass spectrometry and database search (Table 1).

### 2.4. Analysis of Spot Volumes

**Figure 4.**
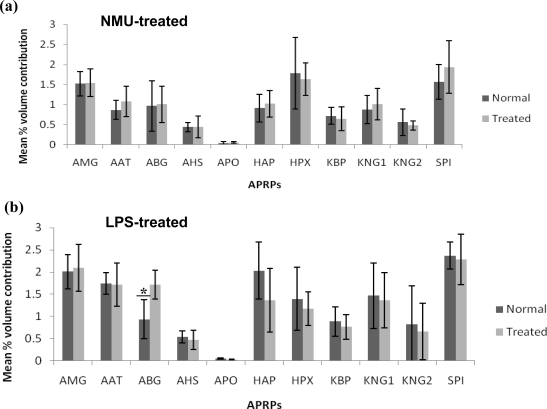
Mean percentage of volume contribution of rat serum APRPs. Panels demonstrate values for rats induced with (**a**) breast cancer and (**b**) inflammation, *versus* their respective control sera. Analysis was determined using the ImageMaster^TM^ 2D Platinum Software 7.0. A *p* value of less than 0.05 was considered significant (*).

Volume analysis of the 13 spot clusters within and between the different groups of rat serum samples demonstrated comparable expression of almost all the highly abundant proteins detected. For most protein clusters, comparable expression was detected between the groups of rats treated with NMU compared with those of the rat normal serum samples (Figure 4a). The spot clusters of albumin and fetuin-B precursor were apparently not conducive to the analysis. In contrast, the group of rats injected with LPS showed significant variation in the expression of alpha-1B glycoprotein (Figure 4b).

## 3. Discussion

By 18 weeks post injection of NMU, none of the rats in the breast cancer group demonstrated any signs or symptoms of the disease on general observation or palpation, although histopathological examination of the mammary gland tissues showed an indication of early invasive cancerous change. This apparent contradiction may be explained to a certain extent by differing reports in the literature. For example, one report indicated a mean incidence of one cancer per rat at the 16th week after carcinogen administration [20], whereas Roomi *et al.* (2005) observed that the probability of generating palpable tumors in rats is higher at more than 20 weeks subsequent to NMU injections [21].

Despite the lack of physical evidences, marked enhancement of tumor in the mammary gland of rats treated with NMU was successfully detected by contrast enhanced MRI. Most previous imaging studies of the mouse model focused on large mammary tumors and were performed in dedicated animal MRI units of 4.7 Tesla to 7 Tesla. This study showed the possibility of detecting very small amounts of breast cancer tissues using standard clinical 3 Tesla MRI equipment. In addition, the study was performed with just a basic custom made mold to house the rat, a pre-estimated dosage of contrast according to the weight of the rat and imaging within a standard knee coil. However, more cases that correlate to the biopsies need to be performed.

On the contrary, serum proteomic profiles of highly abundant proteins from the control sera and those obtained from sera of rats that were induced with breast cancer at the 18th week after injections with NMU did not show significant differences in the expression of any of the proteins analyzed. This suggests that APRPs at the very early stages of tumor development are not detectable by current proteomic approaches, even if they were present. Apparently, the rat tumor has to reach some critical mass before significant quantities of APRPs enter the circulation.

In contrast to the rats that were chemically induced with breast cancer, those treated with bacterial LPS showed significant up-regulated expression of alpha-1B glycoprotein at the 18th week after receiving the injections. The expression of other APRPs was not altered. Alpha-1B glycoprotein, a serum protein of unknown function, appeared to be part of the first line of APRPs that was induced in response to inflammation. The altered expression of serum alpha-1B glycoprotein in the rats treated with LPS forms an early evidence for the difference in the acute-phase response to inflammation from that which was induced by breast cancer.

When taken together, the data of our study demonstrated the possibility of contrast enhanced MRI recognition of a tumor at an early stage of breast cancer when the APRP changes in the rat sera were not able to illustrate clear differences.

## 4. Experimental Section

### 4.1. Rat Serum Samples

Twelve, two month old, inbred and virgin female Sprague-Dawley rats were used. Each rat was individually caged at the animal quarters at 23–25 °C and 35–55% humidity, with normal light-dark cycle. Regular chow pellets and normal tap water were supplied *ad libitum*. The first bleeding was performed five days prior to treatment with chemicals and the serum samples obtained were used as normal controls. Blood was taken from the tail vein using 23 gauge butterfly needles and serum samples were stored at −80 °C. The rats were sacrificed 18 weeks after the first injection of chemicals and their breast tissues were dissected. All experimental procedures were approved by the Animal Research Committee of the University of Malaya Medical Centre in accordance with the guide for care and use of laboratory animals.

### 4.2. Chemical Treatment

*N*-Methyl-*N*-nitrosourea (NMU) and lipopolysaccharide (LPS) from *E. coli* were purchased from Sigma Aldrich Chemical Company (St Louis, MO, U.S.). Six rats received a single intraperitoneal dose of 50 mg/kg NMU for inducement of breast cancer while the rest received 0.06 mg/kg LPS to initiate inflammation. The NMU solutions were used within 15 min of preparation.

### 4.3. Histological Staining

The abdominal-inguinal and contralateral mammary glands were collected from euthanized rats at 18 weeks after the NMU injections. The mammary glands were fixed in 10% formalin overnight, processed and embedded into paraffin blocks. Sections were cut at 4 μm thickness and stained with Hematoxylin (Biostain Ready Reagents Ltd, Cheshire, UK) and Eosin (Richard-Allan Scientific, Kalamazoo, MI, U.S.) on frosted slides. Breast tissues were examined under a light microscope to observe for microscopic cancerous and precancerous lesions and confirmed by an experienced histopathologist.

### 4.4. Contrast Enhanced MRI

Rats at the 14th week after NMU injections were anesthetized by intramuscular injection with ketamine (80–110 mg/kg) and xylazine (12 mg/kg) (Troy Laboratories Australia Pty Ltd, Sydney, Australia), to prevent movement prior to MRI. A 24 gauge angiocatheter was placed in the tail vein and injected with heparin-saline to prevent blockage. An IV contrast agent, gadopentetate dimeglumine (Magnevist—469 mg/mL; Bayer Schering Pharma AG, Berlin, Germany), diluted with heparinized sterile saline solution at 0.2 mL/kg, was injected at the contrast phase of the MRI. The rats were placed on a polystyrene mold that was inserted into the knee coil of a standard clinical 3 Tesla magnet GE Healthcare MRI equipment. A scout image was taken for anatomical references to ensure that the upper and lower mammary glands were covered. T1, T2, and post gadolinium fat sat T1 images were acquired in different planes (see Table 2 for parameter settings).

Although a 30-min sedation for MRI was planned for the rats, scans had to be done within 20 min as some of the rodents developed significant bradycardia and extreme low blood pressure after 15 min. This may be partly due to hypothermia because of the cold MRI room, which may be reduced by using a hot air blower that was directed at the knee coil [22,23]. In a few of the sedated rats, imaging had to be abandoned as contrast was not able to be administered due to constriction of vessels and decreased peripheral blood circulation, especially at the sites for IV contrast administration in the tail vein.

### 4.5. 2-DE and Mass Spectrometry

2-DE and mass spectrometry using the 4800 Plus MALDI TOF/TOF analyzer (Applied Biosystems/MDS Sciex, Toronto, Canada) were performed as previously described [8,24]. Protein spots were identified using the MASCOT search engine (Matrix Science Ltd, London, U.K.; release version 2.1.0) against *rattus* entries in the Swiss-Prot database (Last update October 23, 2008, containing 261513 sequences). The following variables were set as search parameters: trypsin was used; up to one missed cleavage was allowed; variable modifications included were carbamidomethylation of cysteine and oxidation of methionine; the mass tolerance for MS precursor ion and MS/MS fragment ion were 100 ppm and 0.2 Da, respectively; and only monoisotopic masses were included in the search.

### 4.6. Image and Statistical Analyses

The GE ImageMaster^TM^ 2D Platinum Software version 7 was used to analyze expression of proteins in the 2-DE profiles. Percentage volume contribution of protein spots, which refers to the spot volume of a protein expressed as a percentage of the total spot volume of all detected serum proteins, was calculated to detect proteins that were differentially expressed in the rat serum. The ANOVA statistical test was used to analyze the protein spot differences between the profiles. A *p* value of less than 0.05 was considered significant.

## 5. Conclusions

Contrast enhanced MRI using gadolinium as the contrast agent appeared to have the capability to detect the NMU-induced tumor at 14 weeks of development. However, the serum APRP profiles of rats before and 18 weeks subsequent to NMU injections did not show any significant difference, although pathological examination of the breast tissues of the animals demonstrated obvious evidence of early tumor development.

## Figures and Tables

**Table 1.** Tandem mass spectrometric identification of spot clusters from rat serum 2-DE profiles.

**APRP^#^**	**Accession Number (Swiss-Prot)**	**Theoretical Mass**	**Mascot Score**	**Theoretical pI**	**No of Peptide Hits**	**Sequence Coverage (%)**
ALB (serum albumin)	P02770	68686	739	6.46	12	27
AHS (α2-HS glycoprotein)	P24090	37958	263	6.05	4	22
ABG (α1B-glycoprotein)	Q9EPH1	56443	272	6.89	7	14
KNG1 (T-kininogen)	P01048	47745	296	6.08	6	20
KBP (kallikrein-binding protein)	P05545	46532	327	5.31	8	22
SPI-3 (serine protease inhibitor)	P09006	46622	384	5.33	7	21
APO (apolipoprotein A-IV)	P02651	44429	770	5.12	15	45
AMG (α1-macroglobulin)	Q63041	167019	643	6.46	10	6
HAP (haptoglobin)	P06866	38539	508	6.10	12	32
AAT (α1-antitrypsin)	P17475	46107	720	5.70	13	34
HPX (hemopexin)	P20059	51318	498	7.58	9	19
FBP (fetuin-B precursor)	Q9QX79	41506	451	6.71	9	40
KNG2 (T-kininogen)	P01048	47745	424	6.08	8	18

# APRP spot ID are as in Figure 1.

**Table 2.** Parameters used for contrast enhanced MRI. Scanner type: 3.0T Signa Hdxt. Type of head coil: HD T/R knee/foot coil by INVIVO.

**Parameters**	**AX FSE PD**	**AX T1 SE FAT SAT**	**AX T1 SE FAT SAT + CONTRAST**	**SAG T1 FAT SAT + CONTRAST**
**BANDWITH:**	62.5	11.9	11.9	11.9
**FOV**	8 × 8	8 × 8	8 × 8	8 × 8
**FREQ:**	512 × 224	192 × 160	192 × 160	192 × 160
**IMAGING OPTIONS**	FAST, NPW, TRF	NPW, ZIP512, StFCl	NPW, ZIP512, StFCl	NPW, ZIP512, StFCl
**MODE:**	2D	2D	2D	2D
**NEX:**	1	2	2	1
**PULSE**	SEQ:FSE-XL	SEQ:FSE-XL	FSE-XL	FSE-XL
**SHIM:**	AUTO	AUTO	AUTO	AUTO
**SLICE THICK:**	2	2	2	3
**SPACING:**	0	0	0	0
**TE:**	33.5	13	13	13
**TR:**	7660	900	900	740

## References

[1] Ferlay J, Shin H, Bray F, Forman D, Mathers C, Parkin D (2010). Estimates of worldwide burden of cancer in 2008: GLOBOCAN 2008. Int J Cancer.

[2] Jansen SA, Conzen SD, Fan X, Krausz T, Zamora M, Foxley S, River J, Newstead GM, Karczmar GS (2008). Detection of *in situ* mammary cancer in a transgenic mouse model: In vitro and in vivo MRI studies demonstrate histopathologic correlation. Phys. Med. Biol.

[3] Frangioni J (2008). New technologies for human cancer imaging. J. Clin. Oncol.

[4] Hara N, Okuizumi M, Koike H, Kawaguchi M, Bilim V (2005). Dynamic contrast-enhanced magnetic resonance imaging (DCE-MRI) is a useful modality for the precise detection and staging of early prostate cancer. Prostate.

[5] Turnbull L (2009). Dynamic contrast-enhanced MRI in the diagnosis and management of breast cancer. NMR Biomed.

[6] Turetschek K, Huber S, Floyd E, Helbich T, Roberts TP, Shames DM, Tarlo KS, Wendland MF, Brasch RC (2001). MR imaging characterization of microvessels in experimental breast tumors by using a particulate contrast agent with histopathologic correlation. Radiology.

[7] Suffredini A, Fantuzzi G, Badolato R, Oppenheim J, O’Grady N (1999). New insights into the biology of the acute phase response. J. Clin. Immunol.

[8] Doustjalali SR, Yusof R, Yip CH, Looi LM, Pillay B, Hashim OH (2004). Aberrant expression of acute-phase reactant proteins in sera and breast lesions of patients with malignant and benign breast tumors. Electrophoresis.

[9] Doustjalali SR, Yusof R, Govindasamy G, Bustam AZ, Pillay B, Hashim OH (2006). Patients with nasopharyngeal carcinoma demonstrate enhanced serum and tissue ceruloplasmin expression. J. Med. Invest.

[10] Abdul-Rahman PS, Lim BK, Hashim OH (2007). Expression of high-abundance proteins in sera of patients with endometrial and cervical cancers: Analysis using 2-DE with silver staining and lectin detection methods. Electrophoresis.

[11] Chen Y, Lim BK, Peh SC, Abdul-Rahman PS, Hashim OH (2008). Profiling of serum and tissue high abundance acute-phase proteins of patients with epithelial and germ line ovarian carcinoma. Proteome Sci.

[12] Ahmed N, Barker G, Oliva KT, Hoffmann P, Riley C, Reeve S, Smith AI, Kemp BE, Quinn MA, Rice GE (2004). Proteomic-based identification of haptoglobin-1 precursor as a novel circulating biomarker of ovarian cancer. Br. J. Cancer.

[13] Koomen J, Shih L, Coombes K, Li D, Xiao L, Fidler I, Abbruzzese J, Kobayashi R (2005). Plasma protein profiling for diagnosis of pancreatic cancer reveals the presence of host response proteins. Clin. Cancer Res.

[14] Yu K, Rustgi A, Blair I (2005). Characterization of proteins in human pancreatic cancer serum using differential gel electrophoresis and tandem mass spectrometry. J. Proteome Res.

[15] Dowling P, O’Driscoll L, Meleady P, Henry M, Roy S, Ballot J, Moriarty M, Crown J, Clynes M (2007). 2-D difference gel electrophoresis of the lung squamous cell carcinoma *versus* normal sera demonstrates consistent alterations in the levels of ten specific proteins. Electrophoresis.

[16] Pang WW, Abdul-Rahman PS, Wan-Ibrahim WI, Hashim OH (2010). Can the acute-phase reactant proteins be used as cancer biomarkers?. Int. J. Biol. Markers.

[17] Chen Y, Lim BK, Hashim OH (2009). Different altered stage correlative expression of high abundance acute-phase proteins in sera of patients with epithelial ovarian carcinoma. J. Hematol. Oncol.

[18] Murray TJ, Ucci AA, Maffini MV, Sonnenschein C, Soto AM (2009). Histological analysis of low dose NMU effects in the rat mammary gland. BMC Cancer.

[19] Haynes P, Miller I, Aebersold R, Gemeiner M, Eberini I, Lovati M, Manzoni C, Vignati M, Gianazza E (1998). Proteins of rat serum: I. establishing a reference two-dimensional electrophoresis map by immunodetection and microbore high performance liquid chromatography-electrospray mass spectrometry. Electrophoresis.

[20] McCormick DL, Adamowski CB, Fiks A, Moon RC (1981). Lifetime dose-response relationships for mammary tumor induction by a single administration of *N*-methyl-N-nitrosourea. Cancer Res.

[21] Roomi MW, Roomi NW, Ivanov V, Kalinovsky T, Niedzwiecki A, Rath M (2005). Modulation of *N*-methyl-*N*-nitrosourea induced mammary tumors in Sprague-Dawley rats by combination of lysine, proline, arginine, ascorbic acid and green tea extract. Breast Cancer Res.

[22] Schepkin VD, Ross BD, Chenevert TL, Rehemtulla A, Sharma S, Kumar M, Stojanovska J (2005). Sodium magnetic resonance imaging of chemotherapeutic response in a rat glioma. Magn. Reson. Med.

[23] Driehuys B, Nouls J, Badea A, Bucholz E, Ghaghada K, Petiet A, Hedlund LW (2008). Small animal imaging with magnetic resonance microscopy. ILAR J.

[24] Seriramalu R, Pang WW, Jayapalan JJ, Mohamed E, Abdul-Rahman PS, Bustam AZ, Khoo AS, Hashim OH (2010). Application of champedak mannose-binding lectin in the glycoproteomic profiling of serum samples unmasks reduced expression of alpha-2 macroglobulin and complement factor B in patients with nasopharyngeal carcinoma. Electrophoresis.

